# Mitochondrial DNA Mutations Regulate Metastasis of Human Breast Cancer Cells

**DOI:** 10.1371/journal.pone.0023401

**Published:** 2011-08-11

**Authors:** Hirotake Imanishi, Keisuke Hattori, Reiko Wada, Kaori Ishikawa, Sayaka Fukuda, Keizo Takenaga, Kazuto Nakada, Jun-Ichi Hayashi

**Affiliations:** 1 Graduate School of Life and Environmental Sciences, University of Tsukuba, Tsukuba, Ibaraki, Japan; 2 Shimane University Faculty of Medicine, Izumo, Shimane, Japan; Health Canada, Canada

## Abstract

Mutations in mitochondrial DNA (mtDNA) might contribute to expression of the tumor phenotypes, such as metastatic potential, as well as to aging phenotypes and to clinical phenotypes of mitochondrial diseases by induction of mitochondrial respiration defects and the resultant overproduction of reactive oxygen species (ROS). To test whether mtDNA mutations mediate metastatic pathways in highly metastatic human tumor cells, we used human breast carcinoma MDA-MB-231 cells, which simultaneously expressed a highly metastatic potential, mitochondrial respiration defects, and ROS overproduction. Since mitochondrial respiratory function is controlled by both mtDNA and nuclear DNA, it is possible that nuclear DNA mutations contribute to the mitochondrial respiration defects and the highly metastatic potential found in MDA-MB-231 cells. To examine this possibility, we carried out mtDNA replacement of MDA-MB-231 cells by normal human mtDNA. For the complete mtDNA replacement, first we isolated mtDNA-less (ρ^0^) MDA-MB-231 cells, and then introduced normal human mtDNA into the ρ^0^ MDA-MB-231 cells, and isolated trans-mitochondrial cells (cybrids) carrying nuclear DNA from MDA-MB-231 cells and mtDNA from a normal subject. The normal mtDNA transfer simultaneously induced restoration of mitochondrial respiratory function and suppression of the highly metastatic potential expressed in MDA-MB-231 cells, but did not suppress ROS overproduction. These observations suggest that mitochondrial respiration defects observed in MDA-MB-231 cells are caused by mutations in mtDNA but not in nuclear DNA, and are responsible for expression of the high metastatic potential without using ROS-mediated pathways. Thus, human tumor cells possess an mtDNA-mediated metastatic pathway that is required for expression of the highly metastatic potential in the absence of ROS production.

## Introduction

Mitochondrial respiration defects caused by mitochondrial DNA (mtDNA) mutations have been proposed to contribute to tumor development based on evidence that most chemical carcinogens bind more readily to mtDNA than to nuclear DNA [1], [2], somatic mtDNA mutations accumulate at a faster rate in tumor cells than in normal cells [3]–[6], and that some of them induce respiration defects [7]. Mitochondrial respiration defects can probably induce pseudo-hypoxic pathways under normoxia (the Warburg effect) and regulate tumor development [8]–[11]. In contrast, our previous studies using transmitochondrial cybrids obtained by the exchange of mtDNA between normal and tumor cells led us to propose that nuclear DNA, but not mtDNA, controls the transformation of normal cells to develop tumors [12]–[15]. Subsequently, we demonstrated using highly metastatic mouse tumor cells that specific mtDNA mutations that induce mitochondrial respiration defects and overproduction of reactive oxygen species (ROS) can control the malignant transformation of tumor cells to develop the metastatic potential, but do not control the transformation of normal cells to develop tumors [16]. This study applied mtDNA transfer technology to highly metastatic human tumor cells, and showed a pathway that induces metastasis through mtDNA mutation–mediated respiration defects.

## Materials and Methods

### Cell lines and cell culture

The mtDNA-less (ρ^0^) HeLa cells and HeLamtFt cells were established in our previous work [17], and these cells are available in collaboration with us. High metastatic MDA-MB-231 cells were kindly provided by Dr. K. Takenaga (Shimane University Faculty of Medicine, Japan). All the cell lines and the transmitochondrial cybrids listed in Table 1 were grown in DMEM (Sigma, St. Louis, MO, USA) containing 10% fetal calf serum, uridine (50 mg/ml), and pyruvate (0.1 mg/ml).

**Table 1:** Identification of the candidate pathogenic mtDNA mutations that induce complex I defects in MDA-MB-231 cells.

Gene	n.p.	Reference sequence	MCF-7	MDA-MB-231	Amino Acid change
*COXI*	6221	T	-	C	-
*COXI*	6371	C	-	T	-
*ATP8*	8506	T	-	C	-
***ND4***	**12084**	**C**	**-**	**T**	**S442F**
***ND5***	**13966**	**A**	**-**	**G**	**T544A**
*ND6*	14470	T	-	C	-
*Cyt.b*	15310	T	-	C	-

Nucleotide sequence data reported are available in the DDBJ/EMBL/GenBank databases under the Database ID: AC_000021.2 (Reference sequence; Mitomap), AB626609 (MDA-MB-231) and AB626610 (MCF-7).

### Isolation of transmitochondrial cybrids

We isolated mtDNA-less (ρ^0^) MDA-MB-231 by long-term treatment of MDA-MB-231 cells with ethidium bromide. Complete depletion of mtDNA was confirmed by PCR analysis. Enucleated cells of the mtDNA donor were prepared by their pretreatment with cytochalasin B (10 µg/ml) for 10 min and centrifugation at 12,000×*g* for 30 min. Resultant cytoplasts were fused with ρ^0^ MDA-MB-231 cells by polyethylene glycol. Transmitochondrial cybrids were isolated in the selection medium that allows exclusive growth of the cybrids (see Table 2).

**Table 2:** Genetic characteristics of genome donors and selection of the trans-mitochondrial cybrids.

Cells	Nuclear	mtDNA	Fusion combination	Selection
	genotypes	genotypes	Nuclear donors×mtDNA donors	
Nuclear donors^a^				
ρ^0^ MDA-MB-231	MDA-MB-231, HAT^r^	mtDNA less		
mtDNA donors^a^				
HeLamt231	HeLa, HAT^s^	MDA-MB-231	ρ^?^ HeLa×en^b^ MDA-MB-231	6-tg+UP^−^
HeLamtFt	HeLa, HAT^s^	wild type	ρ^0^ HeLa×en fetal fibroblasts	6-tg+UP^−^
Transmitochondrial cybrids^c^				
231mt231	MDA-MB-231, HAT^r^	MDA-MB-231	ρ^?^ MDA-MB-231×en HeLamt231	HAT+UP^−^
231mtFt	MDA-MB-231, HAT^r^	wild type	ρ^?^ MDA-MB-231×en HeLamtFt	HAT+UP^−^

a ρ^0^ MDA-MB-231 cells were used as nuclear DNA donors and mtDNA recipients. As mtDNA donors, we used HeLamt231 and HeLamtFt cybrids sharing the HeLa nuclear genome background to exclude the influence of variations in nuclear-coded cytoplasmic factors on the phenotypes. HeLamt231 cybrids carrying nuclear DNA from HeLa cells and mtDNA from MDA-MB-231 cells were isolated by the fusion of ρ∼ HeLa cells with enucleated MDA-MB-231 cells and subsequent cultivation in selection medium with 6-thioguanine medium without uridine and pyruvate (6-tg+UP^−^). The 6-tg eliminates unenucleated MDA-MB-231 cells and UP^−^ eliminates unfused ρ^0^ HeLa cells, which require uridine and pyruvate due to their complete respiration defects caused by mtDNA depletion. HeLamtFt cybrids carrying nuclear DNA from HeLa cells and mtDNA from human fetal skin fibroblasts were obtained previously [17] by the fusion of ρ^0^ HeLa cells with enucleated human fetal skin fibroblasts and subsequent cultivation in the 6-tg+UP^−^ medium.

b en represents enucleated.

c The mtDNA donors sharing the HeLa nuclear genome background and expressing 6-tg resistance cannot survive in the presence of hypoxanthine/aminopterin/thymidine (HAT). Nuclear donor ρ^0^ MDA-MB-231 cells can grow in the HAT selection medium, but not in UP^-^ selection medium. Thus, only transmitochondrial cybrids can survive in the selection medium.

### Genotyping of mtDNA

For recognition of the C12084T mutation, a 146 bp-fragment containing the 12,084 site was amplified by PCR. The nucleotide sequences from n.p. 12,054 to 12,083 (GAGAAAACACCCTCATGTTCATACAAATcT, small letters indicate the mismatch site) and n.p. 12,199 to 12,176 (CTGTTGTCTCCGAATGCTGGGGAA) were used as oligonucleotide primers. Combination of the PCR-generated mutation with the C12084T mutation creates a restriction site for *Ear* I, and generates 113-bp and 33-bp fragments on *Ear* I digestion. The restriction fragments were separated in 3% agarose gel.

### Assays of metastatic potential

To test experimental metastatic potential, 1×10^5^ cells/100 µl PBS were injected into the tail vein of 6-week-old male BALB/cAJcl-nu/nu mice (CLEA Japan, Tokyo, Japan). The mice were sacrificed 60 days later, and their lungs were removed. The lungs were fixed in the Bouin's solution, and parietal nodules were conducted blind at least two times. The mice were cared for in accordance with the Guide for the Care and Use of Laboratory Animals and experiments were approved by the Animal Care and Use Committee of University of Tsukuba. (Approval number: 10–277).

### Biochemical measurement of respiratory enzyme activities

Mitochondrial respiratory complex I (NADH dehydrogenase), complex II (succinate dehydrogenase), complex III (cytochrome *c* reductase), and complex IV (cytochrome *c* oxidase) are components of the electron transport chain and are located in the mitochondrial inner membrane. The respiratory complexes were assayed as described before [18]. Briefly, for estimation of complex I+III activity, NADH and cytochrome *c* (oxidized form) were used as substrates, and the reduction of cytochrome *c* was monitored by measuring absorbance at 550 nm. For estimation of complexes II+III activity, sodium succinate and cytochrome *c* (oxidized form) were used as substrates, and the reduction of cytochrome *c* was monitored as described above.

### Measurement of ROS production

Reactive oxygen species (ROS) generation was detected with 2′-,7′-dichlorofluorescein diacetate (DCFH-DA; Invitrogen, Carlsbad, CA, USA). Cells were incubated with 5 µM DCFH-DA for 10 min at 37°C in serum-free DMEM, washed twice with Dulbecco's phosphate-buffered saline (DPBS), and then immediately analyzed with a FACScan flow cytometer (Becton Dickinson, Mountain View, CA, USA).

### Measurement of the concentration of lactate in the cell medium

Cells were seeded at 5×10^4^ cells/well of a 6-well plate and cultured for 24 h. The amounts of lactate in the cell medium were estimated using an F-kit _L_-Lactic acid (Roche, Basel, Switzerland).

### Real time PCR

Real time PCR assay was performed on an ABI Prism 7500 Sequence Detection System (Applied Biosystems, Foster City, CA, USA) using QuantiTect SYBR Green PCR Kit (Qiagen, Hilden, Germany). The assay was carried out in a total volume of 25 µl reaction mixture prepared in triplicates in 96-well optical reaction plates or MicroAmp® optical tubes (Applied Biosystems). The amount of cDNA was normalized to that of an internal control, β-actin. The following primers were used: CHIP, 5′-AGGCCAAGCACGACAAGTACAT-3′ and 5′-CTGATCTTGCCACACAGGTAGT-3′; HIF-1α, 5′-CCATTAGAAAGCAGTTCCGC-3′ and 5′-TGGGTAGGAGATGGAGATGC-3′; MCL1, 5′-CGCCAAGGACACAAAG-3′ and 5′-AAGGCACCAAAAGAAATG-3′; and β-actin, 5′-GGTCATCACTATTGGCAACGAG-3′ and 5′-GTCAGCAATGCCTGGGTACA-3′.

### Statistical analysis

The data were analyzed with a Student's *t*-test. All values are the mean ± standard deviation (S.D.), and values with P<0.05 were considered significant.

## Results

### Characterization of highly and non-metastatic human breast cancer cell lines

**Figure 1:**
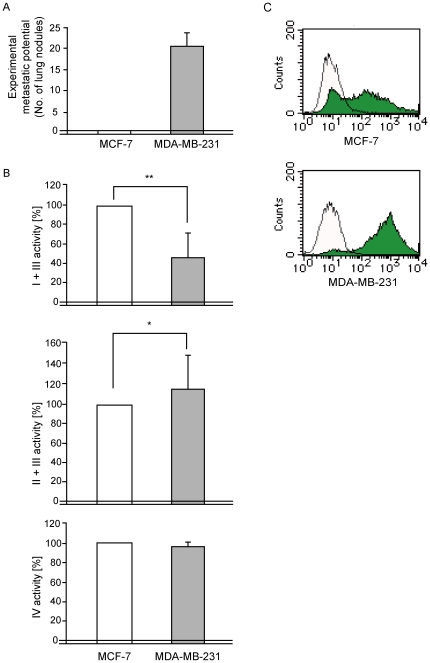
Characterization of human non-metastatic MCF-7 and highly metastatic MDA-MB-231 breast carcinoma cells. (**A**) Experimental metastatic potential. Numbers of lung nodules were counted after inoculation of the cells into the tail vein of nu/nu mice. (**B**) Biochemical analysis of mitochondrial respiratory complex activities. Respiratory complex I, complex II, complex III, and complex IV are components of the electron-transport chain and are located in the mitochondrial inner membrane. Mitochondrial respiratory function was examined by estimating the activities of the complexes. Because the activity of complexes II+III is comparable in MCF-7 and MDA-MB-231 cells, the reduced activity of complexes I+III in MDA-MB-231 cells must result from complex I defects. Bars represent the mean ± S.D. (*n* = 3). *P<0.05; **P<0.01. (**C**) Estimation of the amounts of ROS. Flow-cytometric analysis was carried out using 1×10^6^ cells, which were treated with 5 µM DCFH-DA for quantitative estimation of ROS production.

To examine the effects of mtDNA mutations on the metastatic potential in human tumor cells, we used the human breast carcinoma cell lines MDA-MB-231 and MCF-7, which have high and no metastatic potential, respectively [19]. First, we confirmed their metastatic phenotype by inoculating cells from each line into the tail veins of nu/nu mice and counting the number of metastatic nodules in the lung. As expected, we observed metastasis exclusively in the MDA-MB-231 cells (Fig. 1A).

Our recent study demonstrated that defects in respiratory complex I activity caused by pathogenic mtDNA mutations and resultant overproduction of ROS can be responsible for metastasis in mouse lung carcinoma cells [16]. We therefore compared the activities of respiratory complexes and the amounts of ROS in the human breast carcinoma cell lines. The activity of complex I was reduced in highly metastatic MDA-MB-231 cells (Fig. 1B), which produced higher amounts of ROS than non-metastatic MCF-7 cells (Fig. 1C). Thus, the highly metastatic MDA-MB-231 cells may possess pathogenic mtDNA mutations that could be responsible for the expression of the complex I defects, ROS overproduction, and high metastatic potential.

To identify possible pathogenic mtDNA mutations in the MDA-MB-231 cells, we determined whole mtDNA sequences of MDA-MB-231 and MCF-7 cells, and compared their sequences with the standard human mtDNA sequences (Table 1). The MDA-MB-231 mtDNA showed two missense mutations, C12084T in the *ND4* gene and A13966G in the *ND5* gene. Because both *ND4* and *ND5* encode subunits of respiratory complex I, either or both mutations could be responsible for the reduction of complex I activity in the MDA-MB-231 cells.

### Isolation of transmitochondrial cybrids

Since complex I consists of subunits encoded not only by mtDNA but also by nuclear DNA [20], [21], we cannot exclude the possibility that the complex I defects found in the MDA-MB-231 cells (Fig. 1B) are caused by mutations in nuclear DNA-coded genes. Thus, it is necessary to determine which genome, nuclear or mitochondrial, is responsible for the complex I defects, ROS overproduction, and for the highly metastatic potential expressed in the MDA-MB-231 cells. This issue could be addressed by complete exchange of mtDNAs between the MDA-MB-231 and MCF-7 cells, and by demonstrating that the resultant transmitochondrial cybrids with MCF-7 mtDNA restore complex I activity irrespective of whether their nuclear DNA is derived from MDA-MB-231 cells or from MCF-7 cells.

**Figure 2:**
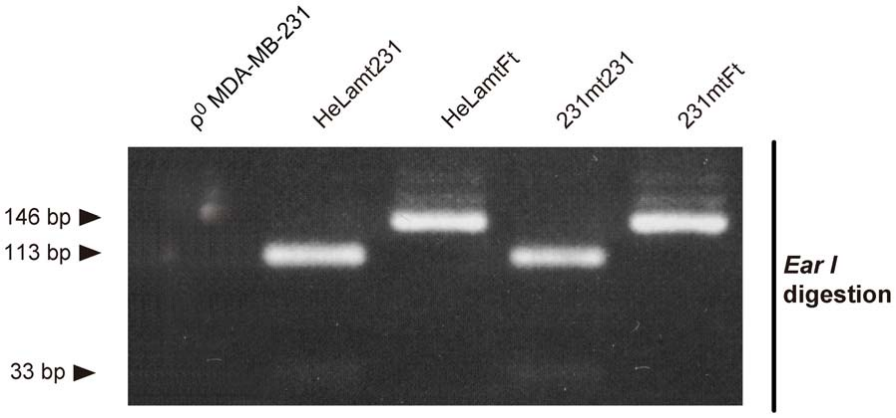
Genotyping of mtDNA in the isolated transmitochondrial cybrids. The ρ^0^ MDA-MB-231 cells have had their mtDNA removed and were used as mtDNA recipients and nuclear DNA donors; HeLamt231 and HeLamtFt correspond to mtDNA donors sharing the HeLa nuclear genome, but possessing mtDNA from the MDA-MB-231 cells and human fetal fibroblasts, respectively (see Table 2). For identification of the C12084T mutation exclusively present in mtDNA of MDA-MB-231 cells (Table 1), the PCR products were digested with *Ear*I. Because of the gain of an *Ear*I site due to the C12084T mutation, 231mt231 cybrids carrying mtDNA from MDA-MB-231 cells produce 113- and 33-bp fragments, whereas 231mtFt cybrids carrying normal mtDNA without the mutation produce a 146-bp fragment.

We tried to obtain mtDNA-less (ρ^0^) cells from the MDA-MB-231 and MCF-7 cells for complete mtDNA exchange and subsequent creation of transmitochondrial cybrids by the fusion of ρ^0^ MDA-MB-231 and ρ^0^ MCF-7 cells with enucleated MCF-7 cells and MDA-MB-231 cells, respectively. However, for unknown reasons we could not obtain ρ^0^ and enucleated MCF-7 cells, although we succeeded in isolating ρ^0^ and enucleated MDA-MB-231 cells. Complete mtDNA depletion in the ρ^0^ MDA-MB-231 cells was confirmed by PCR analysis (Fig. 2).

Thus, instead of transferring MCF-7 mtDNA into ρ^0^ MDA-MB-231 cells, we transferred human fetal mtDNA, which provides normal respiratory function in HeLa cells [17] into the ρ^0^ MDA-MB-231 cells, and obtained transmitochondrial cybrids we named 231mtFt (Table 2). Then, we obtained 231mt231 cybrids, which possess nuclear and mtDNA from MDA-MB-231 cells and thus are genetically equivalent to parental MDA-MB-231 cells, by the transfer of MDA-MB-231 mtDNA into the ρ^0^ MDA-MB-231 cells (Table 2).

### mtDNA genotypes and phenotypes of the cybrids

We performed mtDNA genotyping to confirm mtDNA repopulation in the cybrids. Mismatch primers were designed so that the C12084T mutation exclusively present in MDA-MB-231 mtDNA (Table 1) creates an *Ear*I site in PCR products. As expected, while 231mt231 cybrids gave 113-bp and 33-bp fragments due to the gain of the *Ear*I site in MDA-MB-231 mtDNA, 231mtFt cybrids produced uncut 146-bp fragments (Fig. 2), suggesting that mtDNA had been repopulated properly in both the 231mt231 and 231mtFt cybrids. No PCR products were observed in the ρ^0^ MDA-MB-231 cells due to their mtDNA depletion.

**Figure 3:**
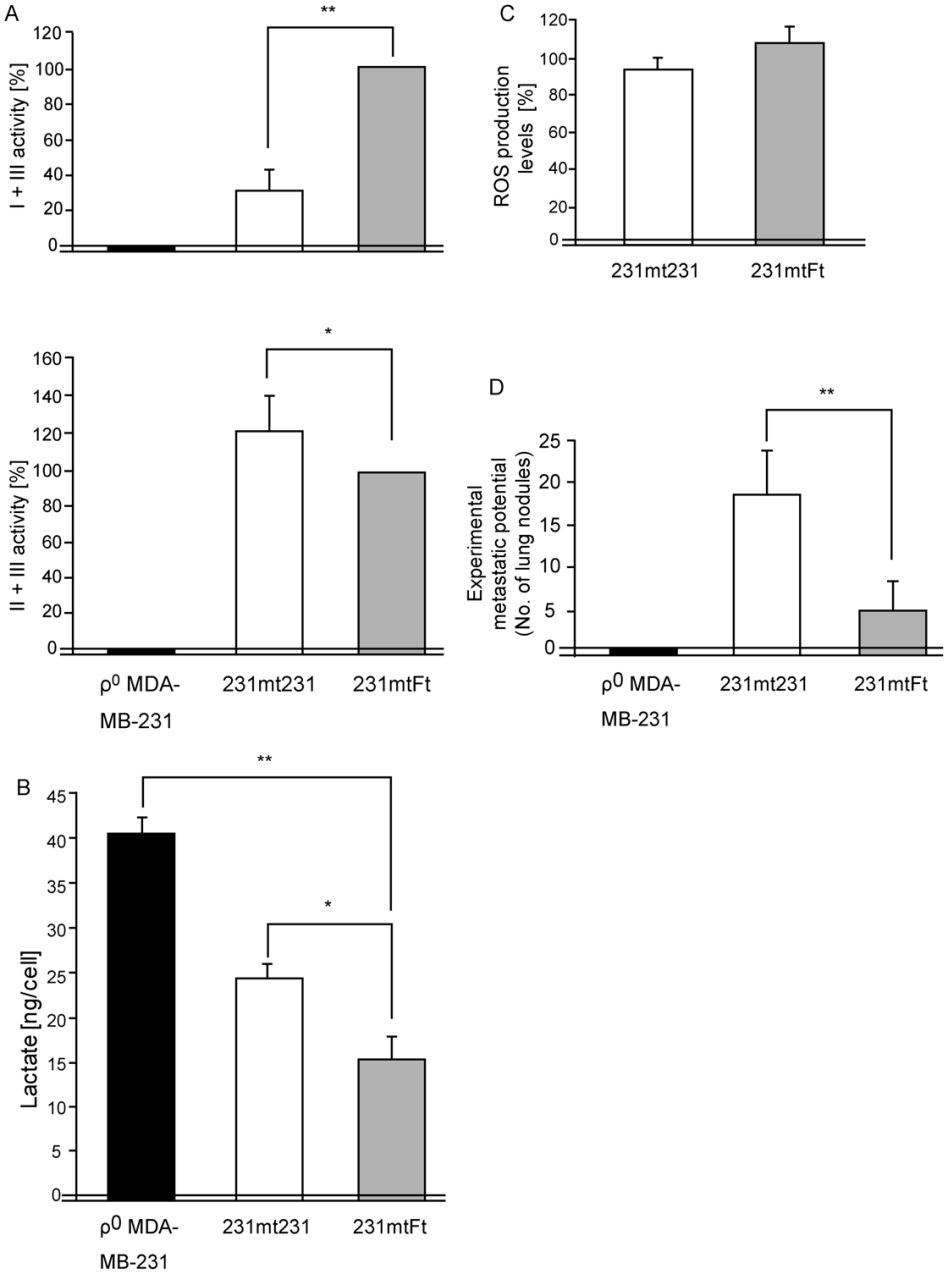
Characterization of the phenotypes of the isolated transmitochondrial cybrids. Using 231mtFt and 231mt231 cybrids, we examined the effects of transferring normal mtDNA from fetal fibroblasts into ρ^0^ MDA-MB-231 on (**A**) mitochondrial respiratory function, (**B**) lactate production, (**C**) ROS production, and (**D**) experimental metastatic potential. Bars represent the mean ± S.D. (*n* = 3). *P<0.05; **P<0.01.

Then, we compared the mitochondrial respiratory function and the levels of lactate and ROS in the transmitochondrial cybrids (Figs. 3A-C). The ρ^0^ MDA-MB-231 cells showed complete mitochondrial respiration defects and enhanced lactate production (Figs. 3A and B), probably due to complete depletion of their own mtDNA (Fig. 2). Complex I activity was higher in the 231mtFt cybrids than in the 231mt231 cybrids (Fig. 3A), and the 231mtFt cybrids showed a slight reduction in lactate level (Fig. 3B). Since these cybrids shared the same MDA-MB-231 nuclear background, the lower complex I activity and higher lactate level in 231mt231 cybrids than in 231mtFt cybrids were due to the mutations in mtDNA of MDA-MB-231 cells (Table 1). However, the ROS level was not reduced in the 231mtFt cybrids (Fig. 3C), suggesting that the mtDNA mutations were not responsible for the ROS overproduction found in the MDA-MB-231 cells (Fig. 1C).

Next, we compared the metastatic potential of the cybrids, and found that the number of the metastatic nodules in the lung was decreased in the 231mtFt cybrids (Fig. 3D). These observations suggest that mtDNA mutations and the resultant complex I defects and lactate overproduction are responsible, at least in part, for the high metastatic potential of MDA-MB-231 cells, and that metastatic potential was suppressed without reduction of the amounts of ROS in the 231mtFt cybrids.

**Figure 4:**
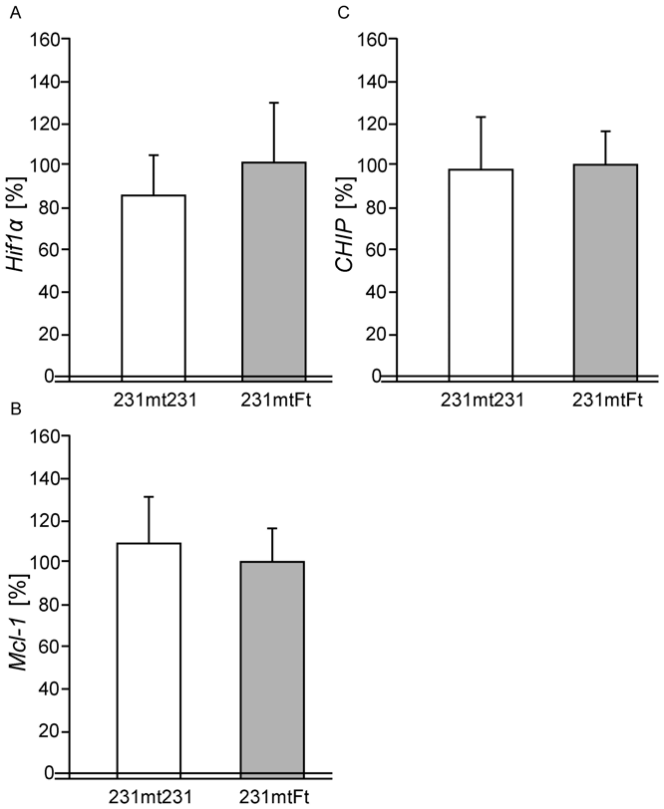
Expression of nuclear-coded genes related to metastasis in the isolated transmitochondrial cybrids. Normal mtDNA from fetal fibroblasts was transferred into ρ^0^ MDA-MB-231 cells, and its effects on the expression of the nuclear-coded genes related to metastasis were examined using real-time PCR analysis (see Materials and Methods). Bars represent the mean ± S.D. (*n* = 3).

We reported previously that mtDNA mutations that induce complex I defects in mouse lung carcinoma cells can increase the metastatic potential via ROS overproduction and resultant overexpression of *Hif1α* and *Mcl-1* [16]. Recently, the downregulation of the U-box-type ubiquitin ligase CHIP, which is encoded by nuclear DNA, has been shown to be responsible for the high metastatic potential in human breast cancer cells [22]. However, the expression of these genes did not change substantially in the cybrids (Fig. 4). Therefore, a pathway besides the ROS- [16] and CHIP-mediated [22] pathways that enhance the metastatic potential is present in high metastatic MDA-MB-231 cells.

## Discussion

We applied mtDNA transfer technology to human breast cancer MDA-MB-231 cells for isolation of transmitochondrial cybrids, and showed that a previously unidentified pathway leading to metastasis, separate from ROS-mediated pathways, is controlled by mtDNA mutations. Moreover, since complete suppression of metastasis was not observed in the 231mtFt cybrids (the transmitochondrial cybrids with normal mtDNA) (Fig. 3D), the high metastasis of MDA-MB-231 cells (Fig. 1A) must be regulated not only by mtDNA mutations, but also by pathways independent of mtDNA, such as the CHIP-mediated pathways regulated by nuclear DNA [22]. Therefore, one of the unresolved issues in this study is how the metastatic pathway in MDA-MB-231 cells is controlled by the mtDNA mutations and regulates metastasis.

It has been proposed that upregulation of glycolysis under normoxia, i.e., the Warburg effect, can be induced by mitochondrial respiration defects and can regulate tumor phenotypes, such as metastatic potential, by the induction of pseudo-hypoxic pathways under normoxia [8]–[11]. We used human breast carcinoma MDA-MB-231 cells and demonstrated that mtDNA mutation–induced mitochondrial complex I defects and the lactate overproduction corresponding to the Warburg effect can be responsible, at least in part, for the metastatic potential. Our previous study [16] also proposed that the complex I defects and the resultant lactate overproduction caused by mtDNA mutations induced metastasis in mouse lung carcinoma cells. However, our subsequent study [23] demonstrated that lactate overproduction is not involved in the metastasis, because metastasis was completely suppressed in the cells expressing the lactate overproduction caused by mtDNA depletion or deletion. Similarly, in the current study the ρ^0^ MDA-MB-231 cells expressing complete respiration defects and lactate overproduction due to mtDNA depletion showed complete suppression of metastasis (Fig. 3D). The apparent discrepancy that metastasis was enhanced by the mtDNA mutations in the 231mt231 cybrids but suppressed by the mtDNA depletion in the ρ^0^ MDA-MB-231 cells (Fig. 3D) may be explained in part by assuming that respiration defects caused by the mtDNA depletion were too severe for the tumor cells to survive. In fact, mtDNA depletion simultaneously induced the loss of respiratory function and the loss of tumorigenicity in ρ^0^ HeLa cells [15].

Therefore, another unresolved issue in this study is whether defects in complex I, but not in other complexes, are required for the expression of metastasis. To examine this idea, transmitochondrial cybrids expressing defects in different respiratory complexes will need to be obtained by introducing mutated mtDNAs derived from patients with various mitochondrial diseases into the ρ^0^ MDA-MB-231 cells.
